# Pyorrhœa Alveolaris—Progress Report

**Published:** 1902-07

**Authors:** 


Pyorrhœa Alveolaris—Progress Report. By Kenneth W. Goadby, D.P.H. (Camb.), L.R.C.P., M.R.C.S., L.D.S. (Eng.).[1]

In a preliminary report made to the Committee, I have stated that the cultural examination had been undertaken in a number of cases and that the organisms obtained had been subcultured on various media, to determine if possible which, if any, of the organisms present were invariably found in all cases; by these means a number of well-known organisms, mostly belonging to the common saprophytes, were found, among them the B. mesentericus ruber and vulgatus. Many members of the yeast family are also found. A culture of one of these isolated from one case produced death when injected into the peritoneal cavity of a guinea-pig, the yeasts were recovered from the peritoneal cavity, but were not present in the blood.

I have, moreover, often found yeasts in the small masses of

[1] Abstract of a paper read before the Odontological Society of Great Britain, April, 1902.

tissue adhering to the apices of teeth extracted in pyorrhœa cases, but as yet these have not been inoculated into animals. Culturally, they appear to correspond to the ones found in the so-called chronic abscesses.

This fact is perhaps interesting when we recall the number of pathogenic members of the Blastomycetes that have come to light in recent years.

Miller, I believe, has described a pathogenic yeast present on one occasion in the mouth, but I have not been able to find its description.

Klein has described a yeast isolated from milk, which produced a general tissue change with the formation of new growths in various parts of the body when inoculated into animals; all of these tumors were filled with yeasts in various stages of growth.

Grasset has also described a pathogenic yeast present in an abscess of the mouth.

Troisier and Achlaime have also described a yeast, pathogenic for guinea-pigs, obtained from the membrane upon the throat of a patient suffering from enteric fever. Fullerton has investigated pathogenic Blastomycetes and describes several varieties, among them Saccharomyces tumefaciens albus which produces tumor formation when injected into animals. The organism described by Grasset appears to be a similar organism to the one I have obtained. It was pathogenic when injected subcutaneously into guinea-pigs. I have frequently met with yeasts of various sorts in the marginal ulceration in children, and in two cases of pyorrhœa in women associated with chronic gastritis and extreme attacks of vomiting. The pathogenicity of these species was not tested.

I have noticed that among the débris from the pus of some of the cases in which these yeasts were shown to be present, there were some large and irregular threads and bacilli which gave the granulose reaction and that in some of the cultures of these yeasts there were present threads and elements of mycelial formation which appeared suggestive of the bacillary forms seen in the specimens stained direct. It is impossible to make any definite statement upon this point at present. The direct examination of the pus furnishes some important points, among which may be mentioned the difference seen in the species of bacteria morphologically represented in early and late cases. It is rare to find many cocci present in the later stages, and the field is, as a rule, occupied by bacilli of various kinds and a good number of threads; among the threads many show a tendency to stain irregularly and in patches, a characteristic that is sometimes met with in the cultivations.

Although cocci are rarely met with in the coverslip direct, they constantly appear if broth cultivations are made from the pus, and among the cocci present the common mouth streptococcus appears with great regularity. Even when the cultivations are made on solid media there is a good deal of difficulty in getting all the organisms seen on the coverslip direct to grow. It does not follow that they cannot be cultivated, but what appears to be the case is that in the mass of organisms are some more or less antagonistic which prevent the growth of each other, probably explaining the curious inconsistence of the species obtained, sometimes the one and sometimes the other organism being able to develop. This, of course, very much complicates the problem, and in addition there are a large number of common saprophytic bacteria of the air that frequently find a lodgement in the mouth. In the cases examined it appears that certain bacteria are generally present and that the others are purely adventitious species, but that of the organisms which are generally present, so far as coverslip indications go, certain ones are very much influenced by the other bacteria developing upon the culture media, so that in some cases they may not grow at all, while in others they are obtained. So far I have been unable to devise a means by which these more exotic bacteria can be always isolated, although I have obtained some of them in pure culture at various times.

By a long process of exclusion certain species have been, I think, excluded from causal relation to pyorrhœa, others appear to be related. One organism, a coccus not hitherto described, may perhaps be mentioned, as it is often present in the pus of dento-alveolar abscesses, where it occurs with tolerable frequency, and appears to have some relation to the curious thick viscid pus one finds in some pyorrhœa cases. This coccus is also found in the pulps of dead teeth associated with abscesses, and especially in acute oral suppuration. It produces a marked stringy viscous growth upon solid media and is extremely difficult to remove from the agar slant. The broth cultures also form stringy viscous growths which at times adhere to the sides of the tube in the form of a cloud of colonies, about 0.5 millimetre in diameter, that are strongly attached to the glass; particularly does this occur in media containing nitrate. This organism will be referred to at a later date.

I have already mentioned the difficulty that there is in obtaining cultivations of all the bacteria that are to be seen on the cover-slip preparations, and it was therefore thought that some additional light on the bacteria of pyorrhœa might be obtained by the direct inoculation of animals with the pus obtained from the pockets.

There is considerable difficulty in obtaining sufficient material from a given case to perform the experiment. Eventually the following method was adopted: After previously wiping the gums with a piece of sterile wool moistened with sterile water, the pockets were cleared out with a sterile instrument and the material obtained mixed up in sterile broth in the form of an emulsion. The emulsion was inoculated into guinea-pigs and in four instances into rabbits. The inoculations were both intraperitoneal and subcutaneous. Dr. Eyre very kindly performed the inoculations for me. The result of these inoculation experiments has given some additional light on the bacteria of pyorrhœa and incidentally on the question of oral sepsis.

Twenty-three animals in all were inoculated, of these nineteen died, some within eighteen hours; the remaining four recovered, but of these two showed local abscess at the site of inoculation, with fluctuation, which cleared up. Several of the animals which succumbed also showed a local abscess which did not burst through the skin, but was gradually absorbed. One guinea-pig showed a local necrosis of the skin. At three of the post-mortem examinations no organisms at all were obtained from the tissues.

In one of the cases the local abscess was incised before death, and the contents showed microscopically the organisms that had been noted on the coverslip; there was a good development on broth and a considerable amount of smell evolved. Plate cultures only gave the mouth streptococcus. In no case were staphylococcus aureus and staphylococcus albus present in the blood or in the tissues and kidney; there was no interstitial abscess formation such as noted by Galippe. The staphylococcus noted above was obtained once from a local abscess at the seat of inoculation, and in one case a streptococcus was obtained from the heart blood. In four cases a streptobacillus tending to form rather long threads of unjointed bacilli was isolated.

The organism in each case seemed to be identical, cultivated on various media it conformed to a general type. The pure cultivations were injected subcutaneously in three guinea-pigs with a positive result. From none of the animals inoculated was the pneumococcus obtained from the heart blood, and no capsuled cocci were observed in the blood. This last is perhaps of interest, as the pneumococcus is frequently present in the saliva, as has been pointed out by Washbourn and Eyre. Kirk has also described a coccus which he thought resembled the pneumococcus as occurring in the abscesses attached to living teeth, and suggested that the infection had arisen from the organisms gaining access through the mucous glands. Kirk, however, does not consider his evidence conclusive.

Bacillus coli was not obtained from any of the post-mortem examinations on the animals inoculated.

These results are too fragmentary to allow a deduction to be made at present, and only serve to illustrate the complicated nature of the problem; at the same time they in no way tend to show that pyorrhœa is a condition generally referable to infection with the common pus cocci. I must say that at first I was inclined to look on the matter rather from this point of view, but the results of my experiments tend to negative that assumption. It may be that the mouth streptococci were in some way accountable for the animals' deaths, as it is well known that the injection of certain organisms tend to promote the pathological effects of other organisms with which they are injected. Thus, for instance, the virulence of attenuated diphtheria bacilli may be increased if injected into an animal together with the streptococcus pyogenes, an attenuated streptococcus pyogenes by injection with the bacillus coli, an attenuated bacillus of malignant œdema by injection with a culture of bacillus prodigiosus, and so on with very many other bacteria. What may have happened, therefore, is that the cocci which often appear on the culture media and do not appear in the post-mortem examinations have so far assisted the pathogenic effect of the other organisms that what would be a non-fatal dose has proved to be a lethal quantity. The cocci to which I refer are the streptococci of the mouth which appear in almost every culture, and which have been obtained from the site of inoculation in certain cases. That some such interaction and symbiosis does take place is probable, but at the same time I think that there is another explanation to be offered of the pathogenic effect of pus.

In several of the inoculations performed there was no fatal issue, although the cases in no way differed from the ones that gave the positive result, and in several of the inoculations the animal did not die for a considerable time; moreover, in four of the fatal cases no organisms whatever were found in the blood or in the tissues; there was no abscess formation; at the site of inoculation there was at first a slight local reaction, which entirely cleared up before the animal's death. Examination of the kidney showed no abscesses, and one animal which did not die was killed, and the kidneys examined and found to be entirely normal, and to show no evidence of the abscess formation which is generally associated with the injection of pus cocci (staphylococci). It therefore occurred to me that the organisms concerned in the process might be of the toxine-forming species, and the following experiment goes some way to show that such a probability is possible. A broth culture from a pyorrhœa case in which gastric trouble had been in evidence for some time was filtered through a Pasteur-Chamberland filter after seven days' incubation at 37.5° C. The filtrate was inoculated into a guinea-pig weighing six hundred and twenty grammes, the amount injected being 4.5 cubic centimetres,—that is, less than one cubic centimetre per one hundred grammes body weight of guinea-pig. The animal died in forty-eight hours.

Cultivations made at the post-mortem examination from the blood and organs gave negative results. There was a gelatinous exudation at the site of inoculation, and the suprarenal capsules were decidedly hemorrhagic. The rest of the organs were normal. Another filtered culture gave a similar result.

Unfortunately, two experiments are not enough to more than point to a probable existence of toxine, but it is at the same time a point of not a little interest, and the following case strongly confirms the supposition of toxine formation by mouth bacteria.

The patient, an insurance agent, consulted his medical adviser (Dr. P. M. Macgregor) complaining of weakness and deep-seated pain in the muscles of his legs and thighs. He had difficulty in walking, but no lightning pains or definite ataxia. He could not sleep, and suffered from severe mental depression. He complained of no gastric symptoms beyond a distaste for food, although his mouth was in an extremely septic condition. He had increased knee-jerks and patches of hyperæsthesia on the feet. The thigh muscles were tender on deep pressure, but there was no pain along the nerve-trunks. There were no thermal changes and no trophic disturbance. There were no eye changes.

There was considerable loss of power to grasp and loss of power in the lower extremities. He was treated for neurasthenia and told to have his mouth attended to, but for some little time refused to have dental attention. Eventually, as he was becoming no better, he consented to submit to dental treatment and consulted me.

There was excessive pyorrhœa with much pus and considerable destruction of the alveolus. The other symptoms were well marked. The affected teeth were removed and mouth lotions prescribed. Improvement immediately set in, the loss of power slowly returning, and within six weeks he was practically well, having lost the chief nerve symptoms, although his grasp was still weak.

In attempting a summary of my investigations the large number of conflicting facts makes the task by no means a light one, and the conclusions may be said to be largely negative, but at the same time there is, perhaps, some additional light on oral sepsis.

It appears highly improbable that the ordinary pus organisms, such as the staphylococci, bacillus pyocyaneus, bacillus coli, etc., have any direct share in the production of pyorrhœa alveolaris.

The absence of the pneumococcus in any of the inoculated animals is also of some interest.

The occurrence of members of the blastomycetes, in both the pus and in the tissues surrounding the roots of pyorrhœa teeth, is instructive and certainly merits further investigation.

Although it is impossible in the present stage of the research to make a definite statement, there appears little doubt that the organisms, whatever they may be, concerned in the process are pathogenic for animals when injected from the gum margin, and that the progress of the disease would appear to be more nearly related to a toxic condition than to the ordinary condition of suppuration.

The relation of pyorrhœa to general toxic conditions and septic gastritis, as pointed out by Hunter, certainly receives confirmation, and the fact of the inoculation of the filtered broth cultivations producing pathogenic effects, points to the presence of toxines that may have much importance in disease.

